# PB.10: Breast compression, compressed breast thickness and volumetric breast density

**DOI:** 10.1186/bcr3511

**Published:** 2013-11-08

**Authors:** J Khan-Perez, C Mercer, M Bydder, J Sergeant, J Morris, A Maxwell, C Rylance, S Astley

**Affiliations:** 1 The University of Manchester Medical School, Manchester, UK; 2 Nightingale Breast Centre and Genesis Prevention Centre, UHSM, Manchester, UK; 3 Centre for Imaging Sciences, Institute of Population Health, University of Manchester, UK; 4 Department of Medical Statistics, University Hospital of South Manchester, Wythenshawe Hospital, Manchester, UK

## Introduction

Adequate compression of the breast during mammography is essential both for ensuring high-quality images and for accurate assessment of breast density using automated volumetric breast density software. Here we explore the relationship between breast density and imaging parameters.

## Method

We identified a set of 210 women undergoing routine screening mammography by the same experienced radiographer using the same mammography unit. Breast density data (breast volume, fibroglandular tissue volume) were obtained using Volpara™ 1.4.0 and imaging parameters (compression force, breast thickness and dose) were extracted from the DICOM headers. Statistical analysis using Spearman's rank-order coefficient was used to examine any existing relationships.

## Results

There were significant positive correlations (*P *< 0.01) between both breast volume and gland volume with compressed breast thickness, X-ray dose and compression force (*P *< 0.05). Volumetric breast density was negatively correlated with compression force in the CC view (*P *< 0.01) and thickness in all views (*P *< 0.01), and breast volume had a significant positive correlation with gland volume (*P *< 0.01).

## Conclusion

Our results show that large, dense breasts had greater thicknesses, higher X-ray doses and required a greater compression force during mammography. There was insufficient evidence to determine whether higher compression forces in larger breasts were due to increased glandular content. The negative correlations with volumetric breast density expressed as the percentage of the breast volume occupied by dense fibroglandular tissue can be explained by the fact that the positive correlations with breast volume were stronger than those with gland volume.

